# Gene Regulatory Programs of NK Cells Show That NCAM1 (CD56) and KIRs Are Controlled by Genetically Polymorphic Distal Regulatory Elements

**DOI:** 10.1002/eji.70142

**Published:** 2026-02-04

**Authors:** Mariam A. Salem, Aditi Varkey, Matthew D. Estrada, Kruthika Sharma, Caprice D. Eisele, Nitin Chakravarti, Dean A. Lee, Aharon G. Freud, Bethany L. Mundy‐Bosse, Patrick L. Collins

**Affiliations:** ^1^ Department of Microbial Infection and Immunity The Ohio State University Columbus Ohio USA; ^2^ Molecular, Cellular and Developmental Biology Graduate Program The Ohio State University Columbus Ohio USA; ^3^ Department of Pathology The Ohio State University Columbus Ohio USA; ^4^ Biomedical Sciences Graduate Program The Ohio State University Columbus Ohio USA; ^5^ Division of Hematologic Malignancies Sidney Kimmel Cancer Center Thomas Jefferson University Philadelphia Pennsylvania USA; ^6^ Department of Pediatrics College of Medicine The Ohio State University Columbus Ohio USA; ^7^ Center For Childhood Cancer Research Abigail Wexner Research Institute at Nationwide Children's Hospital Columbus Ohio USA; ^8^ Division of Hematology Department of Internal Medicine The Ohio State University Columbus Ohio USA

**Keywords:** epigenetics, human, immunology, NK cell, single nuclei ATAC‐seq

## Abstract

Owing to their immunoprotective properties, natural killer (NK) cells are critical for the innate immune response to pathogens, as well as a new wave of cancer immunotherapy that harnesses natural cytotoxicity. We sought to study the genetic and epigenetic drivers behind human‐specific NK cell receptors, so that we can better understand the underlying cellular function. Here, we present a transcriptomic, proteomic (CITE‐seq), and chromatin (single nuclei ATAC‐seq) profiling of human peripheral NK cell subsets, which was then compared with genomic databases. Through integrative multi‐omics, we demonstrate that CD56^bright^ versus CD56^dim^ NK cell subsets have differential distal regulatory element (DRE) landscapes, with fewer accessible DREs in the CD56^dim^ NK cells. We combine our epigenetic data, deposited Hi‐C, and human genetic data to show mechanisms governing the *NCAM1* (encoding CD56) and the killer cell immunoglobulin‐like receptors (KIRs) loci. We identify an *NCAM1* DRE that binds STAT3 in most NK cells, while identifying a genetic cohort that has motifs for binding repressive BLIMP1 at the DRE and resulting in less CD56 expression. Together, our findings reveal novel epigenetic and transcriptomic systems for the regulation of NK cell receptors driving NK cell cytotoxicity and diversity.

## Graphical abstract

A deeper understanding of the natural killer cell gene expression regulation driving NK cell maturation and cytotoxicity could provide critical insights into optimizing NK therapies. Our findings reveal novel enhancers governing NK cell genes for NCAM1/CD56 and cis‐regulation for KIR receptors and clarify existing models of NK cell cis‐accessibility regulation.

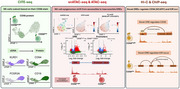

## 1 Introduction

Natural killer (NK) cells play a critical role in the defense against hematological malignancies and infected cells through their unique functions and receptors. Similar to type 1 innate lymphoid cells (ILC1s), which share developmental similarities, NK cells serve as an essential early source of interferon‐gamma (IFN‐γ). However, unlike tissue‐resident ILC1, NK cells are cytotoxic effectors that patrol the blood and lymphatic system. Compared with adaptive CD4 and CD8 lymphocytes, circulating NK cells are unusually short‐lived effectors [1] that depend upon innate recognition. NK cells also differ from their adaptive CD8 counterparts in that they express the killer cell immunoglobulin‐like receptors (KIRs), which allow for missing‐self recognition and the low‐affinity Fc receptor, CD16, that allows antibody‐mediated cellular cytotoxicity (ADCC). Also, unlike conventional T and B lymphocytes, NK cells do not require prior antigen exposure for maturation, allowing their epigenetic programming to be inherently directed toward a committed cytotoxic state.

There are two major phenotypes of circulating NK cells, traditionally identified based upon how brightly they stain for the NK cell‐identifying surface receptor CD56 [2]. Functionally, CD56, encoded by *NCAM1*, is a neural adhesion molecule that also contributes to human NK cell migration [3], development [4], binding to *Aspergillus fumigatus* [5], and Pyk2 signaling [6]. Beyond CD56 function itself, a relatively small change in CD56 staining accounts for a wide range of phenotypes: NK cells that stain brightly for CD56 are termed CD56^bright^, are poorly cytotoxic ex vivo, and show weak or no expression of CD16 and KIRs [7, 8]. In contrast, NK cells with low CD56 staining are named CD56^dim^ NK cells and are highly cytotoxic, express CD16, and express KIR receptors that recognize the lack of self [9, 10]. The CD56^dim^ NK cells also have short telomeres [11, 12], a sign of replicative senescence, and they do not express the high‐affinity cytokine receptor, IL2RA, which is needed for division ex vivo [13, 14].

A deeper understanding of NK cell gene expression regulation is warranted, as uncovering the regulatory mechanisms that drive NK cell maturation and cytotoxicity could provide critical insights into optimizing treatment approaches. To better understand how human NK cell receptors are regulated, we took a chromatin profiling approach, using paired cellular indexing of transcriptomes and epitopes (CITE‐seq) and single nuclei assay for transposase‐accessible chromatin sequencing (snATAC‐seq). Our findings reveal novel enhancers governing NK cell genes for NCAM1/CD56 and cis‐regulation for KIR receptors, as well as clarify existing models of NK cell cis‐accessibility regulation.

## 2 Results

### 2.1 Combined Transcriptomic and Proteomic Profiling Distinguishes NK and ILC Populations

**FIGURE 1:**
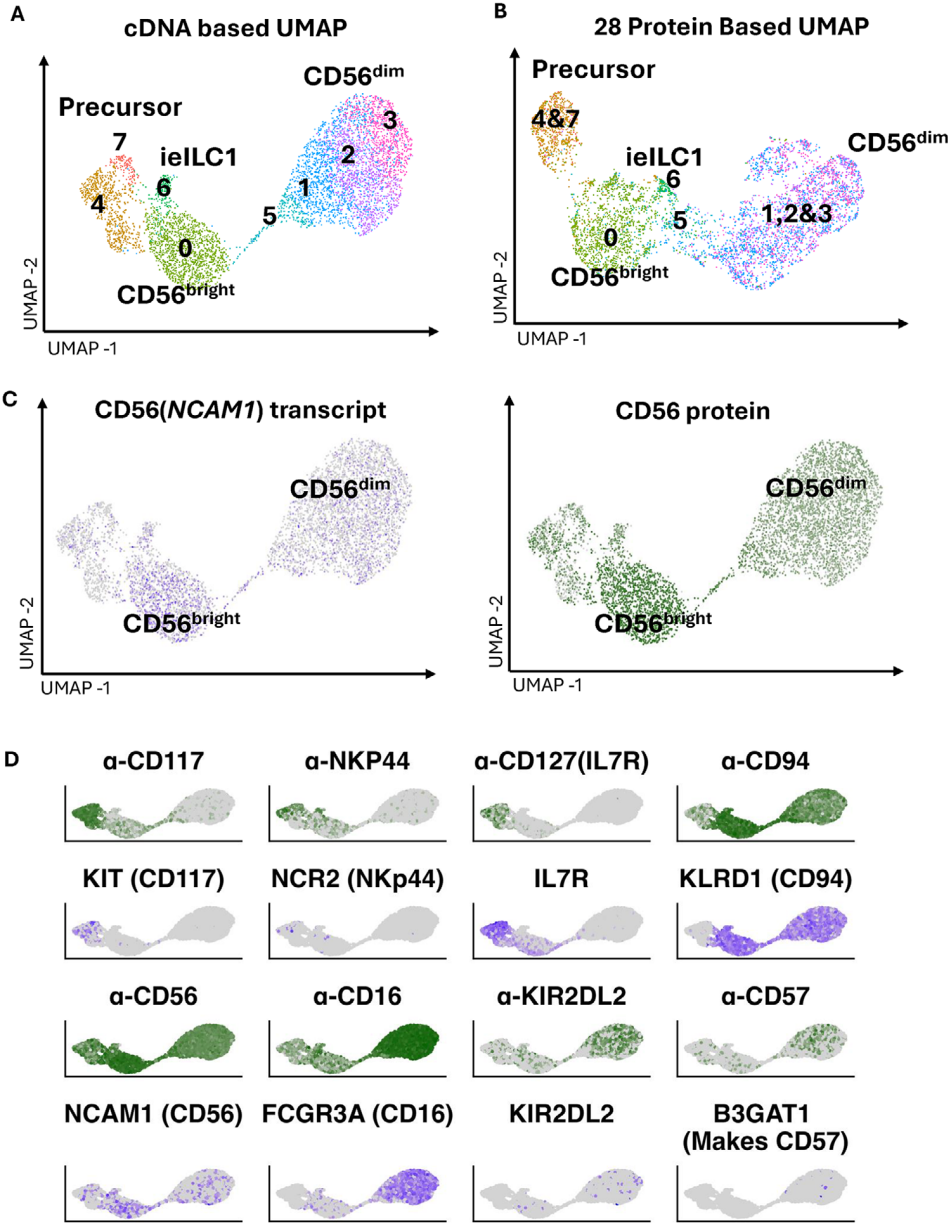
(A) UMAP projection of cDNA derived from peripheral NK cells CITE‐seq. (B) UMAP projection of protein‐based data derived from NK cell CITE‐seq. Clusters are based on the cDNA data (1A) and transferred per cell to the protein UMAP (1B). (C) (left) UMAP plot showing gene transcript level of *NCAM1*. (right) UMAP plot showing the protein level of CD56. (D) (top) Green UMAP plots showing protein and (bottom) purple UMAP showing transcript levels of indicated proteins and genes. In all cases, the darkest color is maximum expression, and grey is no expression.

We initially took a single‐cell‐omics approach to understand the transcriptional programs of human NK cells from healthy blood. We evaluated NK cells from the circulation of 12 healthy donors, as well as NK cells and ILCs from a secondary lymphoid organ (the tonsil). To include all donors and expand data modalities, we subjected the enriched NK cells and tonsil ILC to single‐cell RNA‐seq along with a 28‐panel antibody library, termed CITE‐seq (Figure S1). Resulting expression data were clustered based on the cDNA via hierarchical analysis, which we kept constant between modalities for comparison. When we concurrently embedded cells based upon uniform manifold approximation and projection (UMAP) of cDNA‐ or protein‐based measurements, both the cDNA (Figure 1A) and protein (Figure 1B) data produced similar UMAP plots. The majority of tonsil‐derived ILCs and NK cells were on the left of the plots, and blood‐derived NK cell subsets clustered on the right.

Having protein data allows validation of the transcriptional identification of cell groups. Clusters were initially compared with data for neural cell adhesion molecule 1 (*NCAM1*), which encodes for CD56 (Figure 1C). *NCAM1* transcript and CD56 protein signal were correlated (*p* < 0.05, linear regression) such that CD56^bright^ versus CD56^dim^ NK cells could be clearly identified with either dataset. Figure 1D shows further paired surface marker data (green) and expression pseudo‐colored data (purple). Indeed, generally, clusters with protein staining were usually positive for underlying expression patterns, such as protein anti‐CD117 and transcript KIT, both being observed in clusters 4 and 7, which are ILCs and their precursor cells (Figure 1D).

Cases wherein transcript data were insufficient for cellular identification occurred when gene products were evident in protein data, but the cDNA signal was below reliable detection thresholds. The surface receptor NKp44 was a notable example, which usually marks tonsil‐resident ILCs and NK cells. Anti‐NKp44 antibody‐derived tag (ADT) signal was observable in clusters 4 and 7, but the underlying transcript NCR2 was below dropout levels (Figure 1D). A second example is the marker CD57, which is a terminal carbohydrate epitope (glucuronic acid 3‐sulfate) added to proteins by the glucuronyltransferase *B3GAT1* (also designated GlcAT‐P) [15], and KIR receptors. KIR2DL2 and *B3GAT1* expression levels were too low to discern by transcript, and CD57 or KIR2DL1 ADT reads were required to unambiguously assign CD56^dim^CD57^+^ identity.

### 2.2 Integrating Promoter and Distal Regulatory Element Accessibility to Resolve NK Cell Identity

CITE‐seq analysis revealed that transcript abundance alone is insufficient to infer protein expression. To comprehensively understand the regulatory landscape of NK cells, it is essential to integrate chromatin accessibility and 3D genome topology. Hence, to study the correlation between transcript level, protein levels, and promoter accessibility in NK cells, we performed single nuclei ATAC sequencing (snATAC‐seq) that can be used to predict transcriptional regulators and broader patterns of chromatin regulation. We isolated peripheral NK cells using a negative enrichment rosette [16], with the goal of avoiding activation and downstream changes in chromatin accessibility.

**FIGURE 2:**
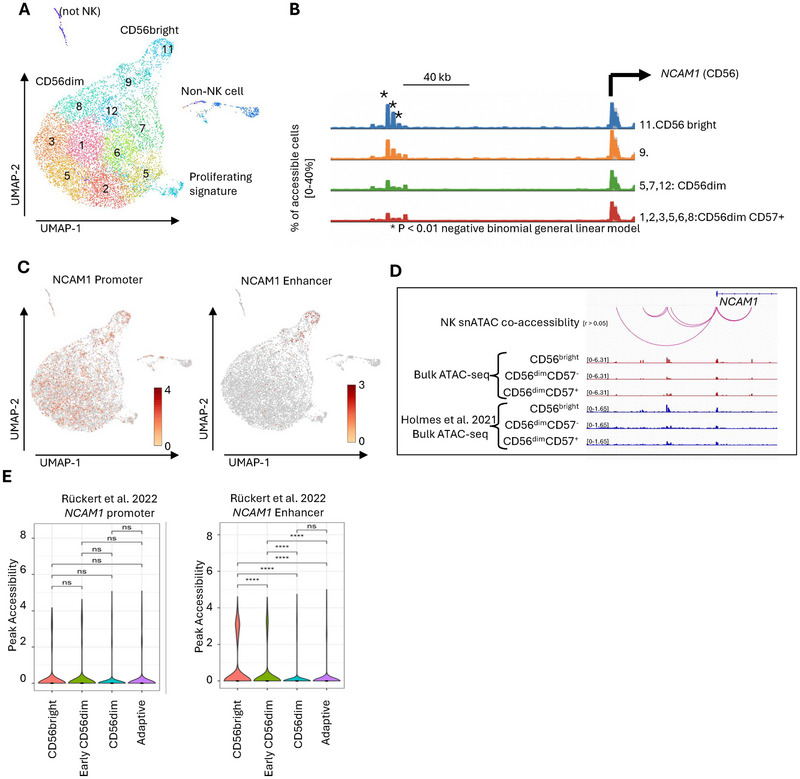
(A) UMAP projection of snATAC‐seq derived from peripheral NK cells. (B) snATAC‐seq tracks of the *NCAM1* locus indicated clusters are on the right in the snapshot. Peaks height indicates percentage of accessible cells in each cluster. (C) (left) UMAP projection of the *NCAM1* promoter accessibility. (right) UMAP projection of accessibility in the regulatory element of the *NCAM1* locus. Colors represent normalized read counts. (D) IGV snapshot of the accessibility of CD56^bright^, CD56^dim^CD57^−^, and CD56^dim^CD57^+^ (red tracks) NK cells from bulk ATAC‐seq at the *NCAM1* locus compared with published bulk ATAC‐seq from Holmes et al. [17] (blue tracks). (E) Accessibility at the *NCAM1* promoter and *NCAM1* DRE compared between CD56^bright^, early CD56^dim^, CD56^dim^, and adaptive NK cells from a published ASAP‐seq from Rückert et al. [18]. *p* < 0.05, **p < 0.01, ***p < 0.001, and ****p < 0.0001.

We initially used promoter accessibility to assign cellular identity to the snATAC‐seq data because we could not unambiguously assign direct protein staining. However, unambiguous identity assignment was challenging with a promoter accessibility‐centric approach (Figure S2A). Within the UMAP embedding, there was a large group of nuclei corresponding to NK cells (Figure 2A). We reasoned that the CD56^bright^ NK cells should have more *NCAM1* promoter accessibility because they express more *NCAM1* transcripts. However, the *NCAM1* promoter was statistically unchanged between different cluster groups in the three different donors (Figure 2B,C; Figure S2F,G). Notably, an upstream region 82 kb from the promoter lost accessibility in the putative CD56^dim^ NK cell clusters (Figure 2B; Figure S2B,D). To validate the accessibility pattern found in the NCAM1 promoter and upstream region, we analyzed published single‐cell assay of transposase‐accessible chromatin (ATAC) with select antigen profiling by sequencing (ASAP‐seq) of human NK cell subsets from peripheral blood of HCMV^−^ donors, as well as published data of bulk ATAC‐seq of human NK cells [17, 18]. In both datasets, we found that indeed the promoter accessibility is unchanged between different NK subsets, CD56^bright^ and CD56^dim^, while the upstream region has more accessibility in the CD56^bright^ NK cells (Figure 2D,E). Therefore, instead of restricting our analysis to promoter accessibility, we also included accessibility of promoters together with linked distal regulatory elements (DREs).

### 2.3 Natural Killer Cell Epigenomes Show a Shift from Accessible DREs to Inaccessible DREs

**FIGURE 3:**
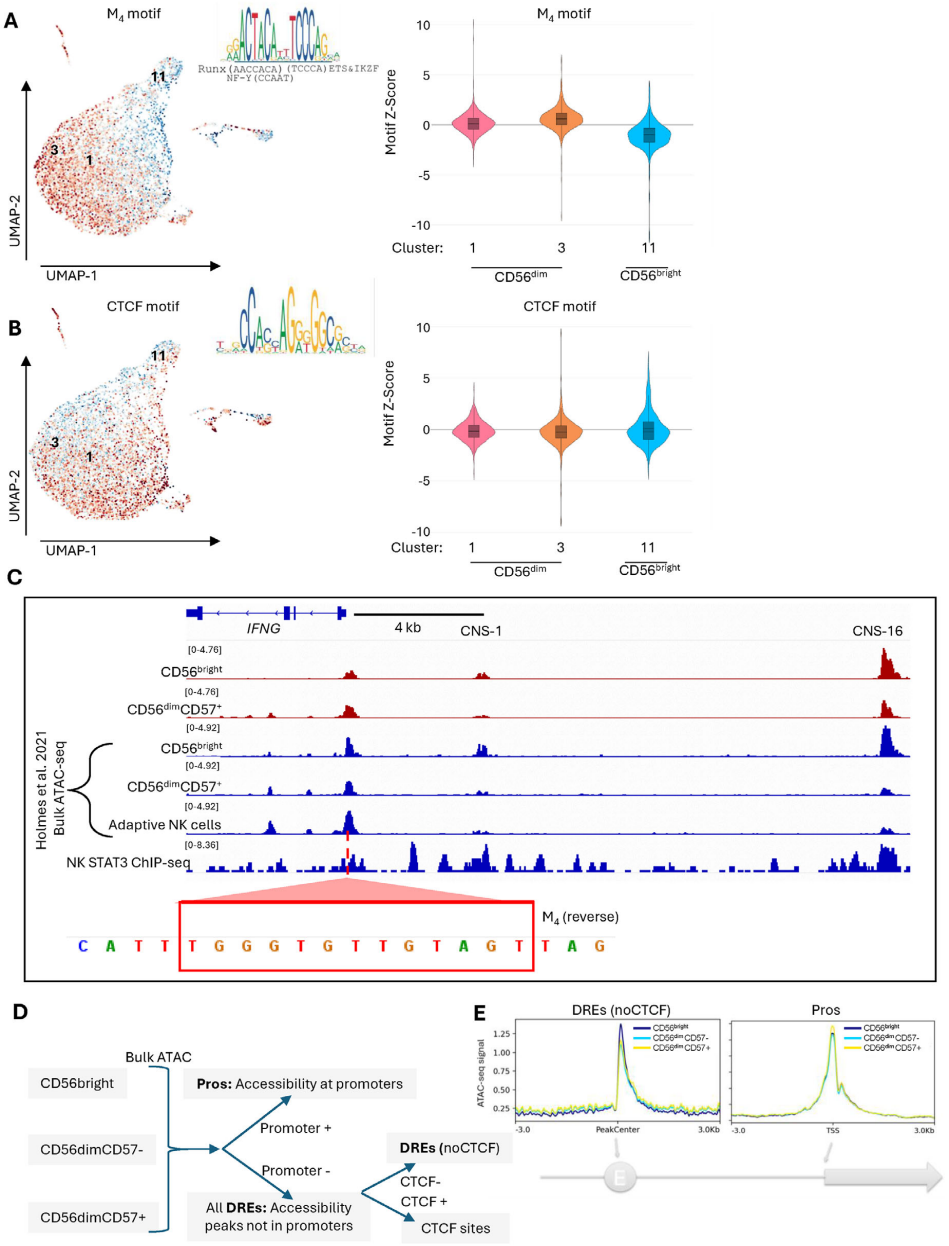
(A) (left) UMAP projection of snATAC‐seq derived from NK cells showing accessibility across all promoters (M_4_ motif) within NK cell snATAC‐seq data. Color represents Z score (red, high; blue, low). (right) volcano plots of accessibility at THAP11 motif (identical to M_4_ motif) in cluster 1, 3, and 11. (B) (left) UMAP projection of accessibility sites with CTCF motif in NK cell snATAC‐seq data. (right) volcano plots of accessibility at CTCF motifs in cluster 1, 3, and 11. (C) IGV snapshot of CD56^bright^ and CD56^dim^CD57^+^ (red tracks) accessibility from bulk ATAC‐seq, as well as accessibility from published bulk ATAC‐seq from Holmes et al. [17] (blue tracks), at the IFNG promoter and distal element. M_4_ motif shown in the promoter region of IFNG. (D) Workflow of bulk ATAC‐seq accessibility analysis, derived from CD56^bright^, CD56^dim^CD57^−^, and CD56^dim^CD57^+^ NK cells. Peaks at promoters were separated from all nonpromoter peaks. Then, nonpromoter peaks were separated based on CTCF peak analysis. (E) Reads were plotted using Bedtools from CD56^bright^ (dark blue), CD56^dim^ CD57^−^ (light blue), and CD56^dim^CD57^+^ (yellow) NK cells, either in distal regulatory elements (left) or promoters (right).

We took an agnostic approach to snATAC‐seq analysis, examining global trends for promoter and DRE accessibility. To analyze global promoter accessibility in NK cells, we utilized the hyperconserved motif in lymphocyte promoters named the M_4_ motif (ACTAYRnnnCCCR) [19]. The M_4_ motif is of interest because it is a combination of RUNX (AACCACA), NF‐Y (CCAAT), and ETS/IKZF1 (TCCCA) motifs. The combined RUNX and ETS/IKZF1 motif highlights the essential role of the motif in lymphocytes, as these are some of the main transcription factors governing lymphocyte transcription. Meanwhile, the NF‐Y motif directs initiation of transcription by binding the transcription initiation complex. The M_4_ motif indeed binds THAP11 and Ikaros factors (IKZF3, IKZF1) directly and NF‐kB indirectly [20, 21]. Hence, we have used the THAP11 motif in our analyses. Globally, snATAC‐seq data showed more M_4_ promoter accessibility in the CD56^dim^ NK cell groups (Figure 3A; Figure S3A,C), and relatively more CTCF accessibility in CD56^bright^ NK cell groups (Figure 3B; Figure S3B,D) that tend to be enriched at DREs. CTCF regulates the 3D structure of chromatin by binding at chromatin loop anchors in distal regulatory elements and topologically associated domain (TAD) borders [22]. To show a specific example of our global analysis, we looked at the *IFNG* locus accessible regions, specifically the *IFNG* promoter and the conserved noncoding sequence 1 (CNS1) and conserved noncoding sequence 16 (CNS16) elements, in CD56^bright^ and CD56^dim^ CD57^+^ NK cells using bulk ATAC‐seq. CNS1 is 4 kb upstream of the *IFNG* promoter in humans and has characterized functions in enhancing *IFNG* production in T helper cells and NK cells by binding transcription factors such as NF‐κB, STATs, and T‐bet [23, 24, 25, 26]. STAT3 ChIP‐seq in naïve NK cells shows mild STAT3 binding at the CNS1 but increased STAT3 binding at the CNS16. We find that CNS16 is also more accessible in the CD56^bright^ compared to the CD56^dim^ NK cells (Figure 3C). CNS16 deletion was shown to significantly decrease human IFN‐γ concentrations in mouse NK cell cultures [24]. The *IFNG* gene promoter has the hyperconserved M_4_ motif we have used in our analyses. We find that the *IFNG* promoter is slightly more accessible in the CD56^dim^ CD57^+^ than in the CD56^bright^ NK cells. However, CD56^dim^ CD57^+^ NK cells have less accessibility in the CNS1 and CNS16 compared with CD56^bright^ NK cells. We also examined the accessibility of CNS1 and CNS16 in adaptive memory‐like NK cells from a published ATAC‐seq dataset [17]. We found that indeed accessibility at the CNS1 and CNS16 is lost in adaptive NK cells (Figure 3C). This is consistent with previous literature showing that CNS1 is hypermethylated in CD57^−^ NKG2C^−^ and loses methylation in HCMV‐specific CD57^+^NKG2C^hi^ expanded and cytokine‐primed NK cells to produce *IFNG* in response to stimulus [26]. NK cell production of IFN‐γ is stimulant‐dependent. CD56^dim^ NK cells are the main NK cell subset to produce IFN‐γ in response to receptor stimulation on target cells. However, CD56^bright^ NK cells are the main IFN‐γ producers in response to exogenous cytokine stimulation [27]. The differential accessibility profile between the different NK subsets could be a factor, among others, of the separate transcription regulatory circuits NK cells employ in response to different stimuli.

To rule out an issue inherent to snATAC‐seq, and to have unambiguous cellular identity, we processed bulk ATAC‐seq data derived from circulating CD56^bright^ NK cells, CD56^dim^ CD57^−^ NK cells, and CD56^dim^ CD57^+^ NK cells to examine the global loss of accessibility in DREs in CD56^dim^ CD57^+^ NK cells. We segregated promoters, DREs, and CTCF sites, then compared read pileup (Figure 3D). Consistent with single nuclei data, CD56^bright^ NK cells had less promoter accessibility than CD56^dim^ CD57^+^ NK cells. The trend was opposite in distal regulatory elements; CD56^bright^ NK cells had more DRE peak abundance than CD56^dim^ NK cells (Figure 3E).

Previously, we and others have used DEseq2 to analyze bulk ATAC‐seq and H3K27ac‐seq data, which were separated into promoters and CTCF‐negative distal regulatory elements. However, DEseq2 performs a normalization of the data before calculating fold change and *p*‐values, which is done under the assumption that the average fold change between datasets should be equal to zero. Our single nuclei and bulk ATAC‐seq data indicated that a second statistical test should be run. Thus, we ran statistical analysis using a student's *T*‐test (Figure S3F, *p*‐values on volcano plots) and did not use DEseq2 to normalize Log2FC values, which together do not assume the datapoint fold changes are centered on zero. Comparing CD56^bright^ NK cells to CD56^dim^CD57^−^ counterparts, we observed that, indeed, most DREs lost accessibility while some promoters gained small but significant levels of accessibility (Figure 3E). A similar trend was observed when comparing CD56^dim^CD57^−^ NK cells to their CD56^dim^CD57^+^ counterparts.

Last, to rule out an issue with accessibility‐only data, we analyzed motif enrichment in H3K27ac^+^ ChIP‐seq peaks from sorted CD56^bright^ NK versus CD56^dim^ NK cells. Again, we observed an enrichment of CTCF motifs in CD56^bright^ NK active regulatory elements, along with enrichment for *PRDM1* motifs (likely indicating suppression) (Figure S3C). Altogether, global trends in bulk and single‐cell accessibility data for NK cells indicate a shift from distal regulation to proximal regulation, which may have been previously unnoticed due to data normalization steps.

### 2.4 NCAM1 (CD56) is Controlled by Distal Regulatory Elements

**FIGURE 4:**
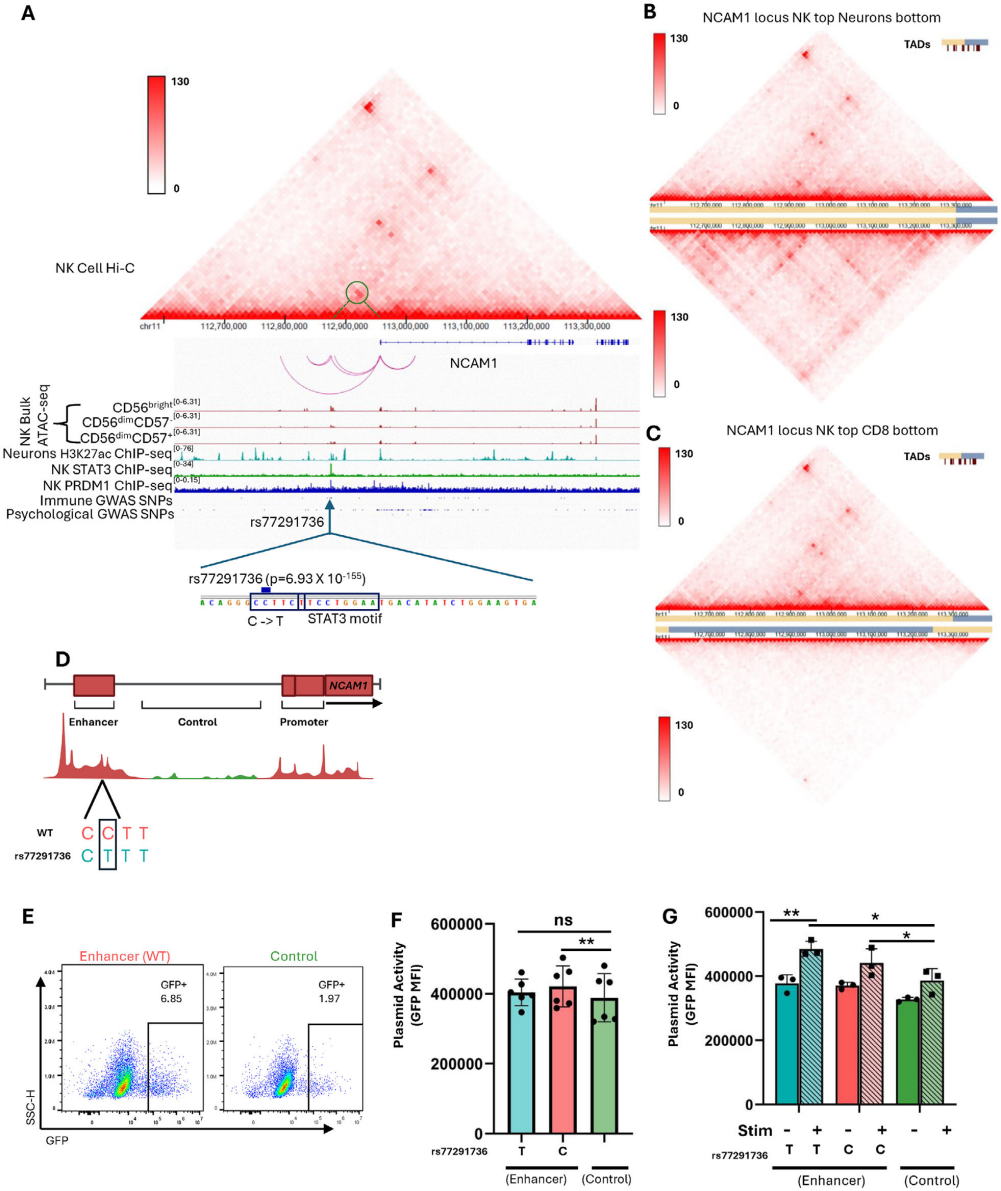
(A) Hi‐C of NK cells (top). IGV snapshot of the *NCAM1* locus (Middle). Upper tracks: cicero co‐accessibility analysis of the *NCAM1* locus, co‐accessibility cutoff was set to higher than 0.05. Middle red tracks: bulk ATAC‐seq from CD56^bright^, CD56^dim^ CD57^−^, and CD56^dim^CD57^+^. Middle blue tracks: H3K27ac ChIP‐seq in Neuronal cells. Middle green track: ChIP‐seq of STAT3 in human NK cells. Middle dark blue track: published ChIP‐seq of PRDM1 in human NK cells. Bottom tracks: GWAS SNPs associated with immunological (top) or psychological (bottom) traits, derived from the GWAS catalogue. (Bottom) Zoomed‐in snapshot of the regulatory element showing the STAT3 motif and rs77291736 SNP. (B) Hi‐C of NK cells (top) compared with neuronal (dorsolateral prefrontal cortex) cells (bottom) in the *NCAM1* locus. Green circles highlight chromatin interactions between the *NCAM1* promoter and the DRE. (C) Hi‐C of NK cells (top) compared with CD8 cells (bottom) in the *NCAM1* locus. Color bars indicate color intensity and are shown to the left. (D) Representative of enhancer region and control region cloned into GFP plasmids for reporter assay. (E) Flow cytometry of viable YTS cells nucleofected with enhancer region (left) and control region (right) upstream of the CMV promoter for GFP. (F) Mean fluorescence intensity of GFP in GFP+ YTS cells nucleofected with enhancer WT, SNP, and control region. (G) Mean fluorescence intensity of GFP in GFP+ YTS cells nucleofected with enhancer WT, SNP, and control region with and without IL15, IL18, IL12, and IL21 stimulus. *p* < 0.05, **p < 0.01, ***p < 0.001, and ****p < 0.0001.

snATAC‐seq co‐accessibility analysis implied that *NCAM1*/CD56 was controlled by loss of accessibility at a cis‐acting element. However, we found the interpretation of snATAC‐seq co‐accessibility difficult because arbitrary algorithm settings could be adjusted, which changes the results. So, to rule out an in silico artifact, we further used bulk ATAC‐seq from FACS‐sorted CD56^bright^ NK and CD56^dim^ NK cell subsets (Figure 4A), middle tracks, data originally from Collins et al. [28]. Indeed, while the *NCAM1* promoter does not change in accessibility according to cell subset in single nuclei ATAC‐seq data (see Figure 2B) or bulk ATAC‐seq (Figure 4A), the newly identified 5’ region does. Since *NCAM1* is highly expressed in neurons, we compared accessibility of the cis‐acting element in neurons with NK cells to understand whether this element is NK‐cell‐specific. The NK cell 5’ DRE has minor acetylation, indicating less accessibility in neurons than in NK cells, showing that it might not regulate neuronal expression of *NCAM1* (Figure 4A). Other than the 5’ regulatory element, our analysis also revealed an intronic element that is accessible only in NK cells, 58 kb downstream from the gene promoter, illustrating multiple possible regulatory inputs.

Chromatin looping is a hallmark of regulatory elements’ function, which enables the proximity of enhancers to their respective gene promoters. Hence, exploring the chromatin topology of the *NCAM1* locus would give us insight into the gene's transcriptional regulation. To evaluate the 3D chromatin topology in NK cells, we utilized ENCODE Hi‐C data of CD56^+^ NK cells [29], their adaptive counterparts, CD8 T cells, and neurons of the dorsolateral prefrontal cortex, where *NCAM1* is ubiquitously expressed. Hi‐C analysis revealed NK cell‐specific DNA interactions between the 5’ cis‐regulatory element and the *NCAM1* promoter (Figure 4A). These chromatin loops were not seen in neuronal cells where the *NCAM1* locus is highly active or CD8 T cells, suggesting that the newly identified regulatory element directly interacts with the *NCAM1* promoter specifically in NK cells (Figure 4B,C; Figure S4A,B). Moreover, we also found that the accessible intronic element within the *NCAM1* gene body forms loops with the gene promoter (Figure 4A).

To test the ability of the DRE to act as an enhancer in NK cells, we nucleofected the NK cell line, YTS, with a construct that has the *NCAM1* DRE upstream of a CMV promoter controlling GFP expression. An equally sized region upstream of the *NCAM1* locus that was inaccessible in NK cells was also inserted upstream of the CMV promoter as a control (Figure 4D). Upon nucleofection, cells with the enhancer had significantly more GFP expression compared with the control, indicating that the *NCAM1* DRE indeed acts as an enhancer in NK cells and can drive increased expression (Figure 4E, F). Our data reveal a previously unrecognized NK‐cell‐specific regulatory element upstream of the *NCAM1* gene that likely shapes CD56 expression through chromatin looping.

### 2.5 SNPs in the CD56 Regulatory Element Affect Protein Levels by Disrupting Transcription Factor Binding

As an additional orthogonal approach to identify what genomic regions control *NCAM1* expression in NK cells, we queried the *NCAM1* regulatory region for naturally occurring human polymorphisms that dictate CD56 protein levels or NK cell immune function. We began with all polymorphisms identified in the region via genome‐wide association studies (GWAS), which we segregated into those involved in neurological or immune conditions. Interestingly, *NCAM1*, as its name, neural cell adhesion molecule 1, implies, is also involved in brain development, and its genomic region was predominantly enriched for disease variants associated with psychiatric and psychological conditions like addiction or depression. We thus proceeded with only immune traits that could be linked to NK cell function for further follow‐up (Figure 4A).

A prior study tested which GWAS polymorphisms change CD56 surface staining levels on natural killer lymphocytes [30] (data extracted and shown in Table S1). To enrich for highly functional variants, they examined an isolated group (Sardinians) [30], which is an approach that decreases genetic diversity and limits differences due to lifestyle and culture [31, 32]. From the Sardinian GWAS study, a series of single‐nuclei polymorphisms (SNPs) overlap with the CD56^bright^ NK cell‐specific accessibility peak we identified through snATAC‐seq co‐accessibility analysis (Figure 4A). Individuals with these SNPs in the newly identified DRE have decreased CD56 surface staining on their NK cells. In YTS cells, we find that the enhancer with the SNP does not increase GFP expression, unlike the WT enhancer without the SNP. Concluding that indeed the SNP decreases overall enhancer function (Figure 4F). Thus, we conclude that the 5’ accessibility region is a genetically validated human *NCAM1* DRE.

To infer the functionality of these DREs, we examined the set of immune‐related polymorphisms for altering transcription factor (TF) DNA binding motifs. Since the genomic block was in a region where genetic variants are inherited together due to linkage disequilibrium, we focused on the SNP with the most significant GWAS p‐value. In that regard, the SNP rs77291736 was most strongly linked to CD56 staining on NK cells in the blood (*p* < 1e‐155, explains 20% of variability) (Figure 4A). Transcription factor binding motif analysis showed that the rs77291736 polymorphism, through a C (most common allele) to T (less common allele) mutation, would create a BLIMP1/*PRDM1* motif (C[T]TTCT) situated next to a STAT3 motif (TTCCNGGAA). In human NK cells, STAT3 is thought to be an activator of proliferation downstream of IL‐21 signaling, while BLIMP1 (encoded by *PRDM1*) is a tumor suppressor that stops the STAT‐dependent cell cycle and enforces cytotoxicity [33, 34, 35]. Thus, as *PRDM1* is thought to be a transcriptional silencer, and STAT3 an activator, such a mutation could be predicted to diminish CD56 expression. While we were unable to find individuals with the rs77291736 allele in our local genetic cohort, we validated that in major allele rs77291736 C/C NK cells, STAT3 binds to the 5’ DRE upon IL‐21 stimuli (Figure 4A, green tracks). We also analyzed published ChIP‐seq of BLIMP1/PRDM1 in NK cells (from SRP512336) and found that it binds the DRE region (Figure 4A, blue tracks). Together, primary human genetic data from an isolated group show that CD56 is controlled by a 5’ regulatory STAT3‐binding region, which is uniquely accessible in CD56^bright^, but not in CD56^dim^ NK cells.

To evaluate how the *NCAM1* DRE, with and without the SNP, responds to STAT stimulus, we stimulated nucleofected YTS cells with a combination of IL12, IL18, IL15, and IL21. Overall, STAT stimulus resulted in a global increase in GFP expression, including in the control construct, likely due to the presence of STAT binding motifs within the CMV promoter. Nonetheless, under STAT stimulus, both the enhancer with and without the SNP drove significantly higher GFP expression compared with the control (Figure 4G), whereas in the absence of STAT stimulus, only the C allele (no BLIMP1/*PRDM1* motif) increased expression. Further, the enhancer containing the SNP exhibited significantly higher GFP expression upon STAT stimulation relative to unstimulated conditions (Figure 4G). These results suggest that the added BLIMP1 (rs77291736 C[T]TTCT) motif does not directly impact adjacent STAT binding in YTS cells, although we acknowledge that transformed NK cell lines such as YTS frequently harbor mutations in STAT3 and *PRDM1*, which may influence the results of the assay [36].

### 2.6 Identification of an NK‐Cell‐Specific Element That Interacts with Multiple KIR Gene Promoters

KIR receptors are crucial for NK viability and the health of humans, as the balance between expression of inhibitory or activating KIR receptors mediates NK cell responsiveness. It has been shown that DNA methylation influences the expression of different KIR alleles [37]. However, the selection of specific KIR expression has garnered significant interest in the context of chromatin topology, particularly regarding the potential roles of specific enhancers and three‐dimensional chromatin architecture in the receptor's selective expression.

**FIGURE 5:**
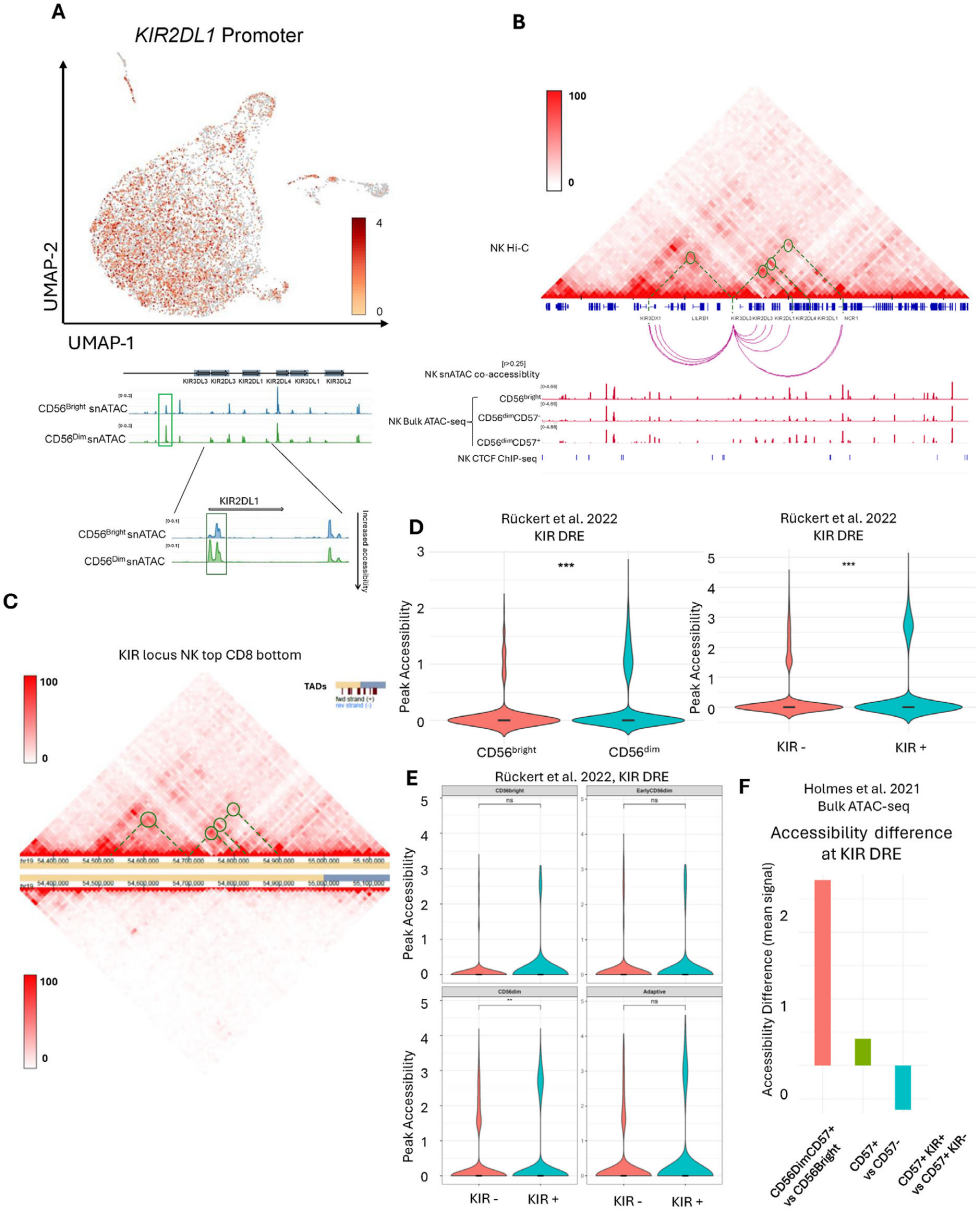
(A) (Top) UMAP projection of snATAC‐seq derived from NK cells in the *KIR2DL1* promoter region. (Bottom) Snapshot showing snATAC‐seq accessibility peaks in the KIR locus in CD56^bright^ and CD56^dim^, and green rectangle around KIR DRE. Lower insert shows the *KIR2DL1* gene region. (B) KIR locus in NK cells. Top Hi‐C heatmap shows NK cells with interactions in the KIR locus. (Bottom) An IGV snapshot of the KIR locus. Upper tracks‐ cicero co‐accessibility analysis of the KIR locus, co‐accessibility cutoff was set to higher than 0.25. Middle red tracks—bulk ATAC‐seq from CD56^bright^, CD56^dim^ CD57^−^, and CD56^dim^CD57^+^ NK cells. Middle blue tracks‐ GWAS SNPs that affect KIR receptor levels. Bottom blue track—ChIP‐seq of CTCF in NK cells. (C) Hi‐C of NK cells (top) compared to CD8 cells (bottom) in the KIR locus. Color bars indicate normalized Hi‐C interaction values. (D) (left) Accessibility at the KIR DRE in CD56^bright^ and CD56^dim^ NK cells from published ASAP‐seq from Rückert et al. [18] (right) Accessibility at the KIR DRE in all KIR‐positive (KIR+) NK cells and all KIR‐negative (KIR−) NK cells from Rückert et al. [18]. (E) Accessibility at the KIR DRE in KIR+ and KIR− NK cells in CD56^bright^, Early CD56^dim^, CD56^dim^, and Adaptive NK cells from Rückert et al. [18]. (F) Mean accessibility difference (*n* = 3) at the KIR DRE between CD56^dim^CD57^+^ and CD56^bright^ NK cells, CD56^dim^CD57^+^ and CD56^dim^ CD57^−^, and CD56^dim^CD57^+^ KIR^+^ and CD56^dim^CD57^+^ KIR^−^ from published bulk ATAC‐seq from Holmes et al. [17]. *p* < 0.05, **p < 0.01, ***p < 0.001, and ****p < 0.0001.

KIR receptors are mostly expressed in the more cytotoxic CD56^dim^ NK cell subset. Our snATAC‐seq analysis revealed that while some KIR gene promoters, such as *KIR2DL1*, are more accessible in the CD56^dim^ NK cell subset, others are not differentially accessible between CD56^dim^ and CD56^bright^ NK cells (Figure 5A). Hence, this indicates that other levels of transcriptional regulation may be in place to govern the expression of KIR genes. Using bulk and snATAC‐seq accessibility, we identified a genomic region of interest that is 76 kb upstream of *KIR2DL1*, which is differentially accessible between CD56^dim^ and CD56^bright^ NK cells (Figure 5A, green rectangle top panel & Figure 5B). This region was specifically more accessible in CD56^dim^ compared with CD56^bright^ NK cells in two other published accessibility datasets (Figure 5D(left),F). To understand whether these peaks are co‐accessible with other KIR gene promoters, we ran a co‐accessibility algorithm on the snATAC‐seq peaks of NK cells, which revealed theorized co‐accessibility with more than one KIR gene promoter (Figure 5B). Since co‐accessibility does not entail DNA interactions, we further used NK cell versus CD8 Hi‐C to test whether this element can form loops with a specific, or several, KIR gene promoter(s) in NK cells. Indeed, Hi‐C in NK cells revealed that there are DNA interactions from the accessible element to several KIR gene promoters that are absent in conventional CD8 T cells (Figure 5B,C; Figure S4C). CTCF ChIP‐seq data from ENCODE (GSE209376) demonstrates that CTCF binding occurs near the boundaries of regulatory element looping (Figure 5B, lower tracks). This suggests that the regulatory element forms loops within a defined topologically associating domain (TAD), selectively interacting with multiple gene promoters while being positioned near CTCF binding sites at the borders [38].

To understand the accessibility of the KIR DRE relation with KIR expression, we analyzed the ASAP‐seq data of human NK cells where “KIR3DL1”, “KIR2DL2‐L3”, and “KIR2DL1‐S1‐S3‐S5” antibodies were used to label NK cells. We created a KIR+ group that is labeled by one or more of the KIR receptors (ADT >1) or a KIR− group that is negative for all three antibodies (ADT>1). We find that the enhancer is significantly more accessible in NK cells labeled with one or more KIR receptors than in NK cells negative for KIR receptor staining (Figure 5D(left),F). Moreover, we also analyzed published bulk ATAC‐seq of presorted NK cell subsets CD56^bright^, CD56^dim^CD57^−^, CD56^dim^CD57^+^KIR^−^, and CD56^dim^CD57^+^KIR^+^. We found that the KIR enhancer is indeed more accessible in CD56dim compared with CD56Bright in three separate donors. We next compared KIR+ and KIR− CD56dimCD57+. We found that the DRE is accessible in both CD56^dim^CD57^+^KIR^−^ and CD56^dim^CD57^+^KIR^+^ and that the KIR+ do not have increased accessibility compared with the KIR− CD56^dim^CD57^+^ (Figure 5F). This could suggest that the accessibility in the DRE precedes KIR expression, which was not revealed at a single‐cell resolution.

## 3 Discussion

To understand the regulation of different NK subsets on both the transcriptomic and proteomic levels, we employed CITE‐seq on human peripheral NK cells. Additionally, we utilized single nuclei and bulk ATAC‐seq as well as Hi‐C to investigate the epigenetic mechanisms governing each NK cell subset. The integration of proteomic, transcriptomic, and epigenetic data from human NK cells uncovered previously unidentified novel regulatory elements and provided new insights into the classification and characterization of NK cells.

We focused our regulatory analysis on the *NCAM1* (CD56) and KIR loci because, while human NK cells are typically identified by CD56 and KIR expression levels for effector status, the underlying cell expression mechanisms cannot be evaluated in model organisms. Combined genetic (published GWAS from rare groups with decreased CD56 expression on NK cells) and genomic analysis (our combined Hi‐C and snATAC‐seq) shows that a distal regulatory element controls CD56^bright^ receptor profile. Normally, it is impossible to fully test human‐specific enhancers in vivo; however, GWAS‐derived SNPs show that a 5’ DRE is critically important in *NCAM1*/CD56 expression. Patients with a minor allele have less CD56 expression on their NK cells. On a molecular level, the polymorphism creates a binding site for the transcriptional repressor BLIMP1, which was also shown by ChIP‐seq to bind the locus. More generally, evaluation of CD56 expression points to the importance of BLIMP1 as a critical suppressor of STAT‐driven NK cell phenotypes and the power of combining isolated group GWAS measurement with co‐accessibility and Hi‐C analysis.

It is unclear if polymorphisms that alter *NCAM1*/CD56 expression will have immunological consequences for known CD56 functions. Functionally, CD56 supports the formation of an NK cell “developmental synapse,” which enables proper signaling and maturation of NK cells in their microenvironment [4]. CD56 also functions as an NK cell pathogen recognition receptor during *A. fumigatus* infection [5]; and through interactions with Pyk2, a tyrosine kinase, CD56 influences the spatial arrangement of components like integrins and activating receptors, and promotes integrin‐mediated adhesion turnover [6].

Expression of the KIR loci is restricted to primates, which co‐evolved alongside HLA‐C, and therefore necessitates human‐centric approaches to understand regulation. KIRs are crucial for regulating the activity of NK cells. The stochastic expression of KIR genes is a unique feature of the KIR system that contributes to the diversity and adaptability of NK cells. This phenomenon ensures that individual NK cells express a random combination of KIRs, which is critical for immune surveillance and maintaining a balance between activation and inhibition. The stochastic regulation of the KIR genes is known to be mediated by bidirectional promoters. These promoters initiate transcription in both forward and reverse directions, influencing multiple gene expression pathways [39, 40]. Novel insights into the genomic organization of the KIR locus identified recombination hotspots and linkage patterns that shape and maintain the diversity of the KIR genes [41]. To gain insight into the mechanisms underlying genetic variation within the KIR locus, which plays a key role in population‐level immune adaptability, we employed Hi‐C and snATAC‐seq techniques. Through this analysis, we identified a new factor possibly contributing to expression variability: chromatin looping. Specifically, distal regulatory elements within the KIR locus and at neighboring genes were found to form loops with KIR gene promoters in NK cells. Using published datasets, we were able to show that the DRE in the KIR locus is significantly accessible in NK cells expressing KIR receptors. Moreover, we believe the DRE is accessible earlier in the CD56^dim^ cells, as its accessibility precedes KIR receptors, as revealed by bulk ATAC‐seq. This reinforces the idea that distal regulatory elements, rather than just proximal promoters, play a crucial role in fine‐tuning KIR receptor levels on NK cells by facilitating selective promoter‐enhancer interactions within the chromatin architecture.

Recent studies have used single‐cell RNA‐seq and snATAC‐seq to further characterize the subsets of NK cells in the blood [42, 43, 44, 45], with some commonalities to this study. For all studies, the initial data embedding appears as a teardrop shape with a small set of cells corresponding to CD56^bright^ NK cells at the point of the drop, and a much larger group of CD56^dim^ NK cells comprising the majority. After embedding, varying clustering algorithms and trajectory imputations are employed to further fractionate the cell groups. For example, a consensus analysis found three clusters of NK cells in blood scRNA‐seq data termed NK2 (CD56^bright^), NK1 (CD56^dim^), and NK3 (CD56^dim^) [43]. The NK1 group has high expression of *GZMB* and *PRF1* that contribute to NK cell cytotoxicity as well as mature NK markers. The NK2 group expression profile was consistent with NK cells in earlier stages of maturity. The NK3 group comprised the NKG2C^+^ adaptive NK cells that expand upon CMV infection via HLA‐E interactions [46] and/or the CD57^+^ senescent NK cells.

Lastly, our study performed a global analysis of peaks in NK cell subsets, which revealed that CD56^bright^ NK cells have more accessible peaks at distant regulatory programs and fewer peaks at promoters, while CD56^dim^ NK cells lose accessibility at distant regulatory elements and retain accessibility in promoters. In our analyses, we used the hyper‐conserved motif in lymphocyte promoters named the M_4_ motif (ACTAYRnnnCCCR) [19]. We show that the CD56^dim^ NK cells are enriched for the M_4_ motif that is a combination of the RUNX (AACCACA) and ETS/IKZF1 (TCCCA) motifs and the initiation motif NF‐Y (CCAAT) between them. Thus, as shown in T and B cells’ gene promoters, we demonstrate that NK cells are also enriched in the combined RUNX, ETS motif [47, 48, 49, 50]. Moreover, this is consistent with the findings that activated NK cells lose accessibility at DREs [51] and more mature NK cells have increased hypermethylation [52]. This supports a model for transcription regulation where distal regulatory programs enable transcriptional control during the CD56^bright^ stage, or at least selectively activate specific loci like the KIR gene cluster. As NK cells mature into differentiated clones and with defined KIR expression, they rely less on distal regulation and exhibit reduced chromatin accessibility. Significant epigenetic changes accompany NK cell differentiation, as shown by in vitro studies, which reveal substantial changes in the transcriptional networks governing NK differentiation and the underlying regulatory mechanisms [53].

## 4 Methods

### 4.1 NK Cell Isolation from Blood Using Rosette Enrichment

Blood samples used were obtained from Versiti Ohio, Red Cross. Natural killer (NK) cells were isolated from peripheral blood mononuclear cells (PBMCs) using the RosetteSep Human NK Cell Enrichment Cocktail (STEMCELL Technologies). Whole blood was first mixed with the RosetteSep NK cell enrichment cocktail, which selectively depletes unwanted cell populations by crosslinking them to red blood cells (RBCs). Following the addition of the cocktail, density gradient centrifugation was performed using Ficoll‐Paque to separate enriched NK cells from the remaining blood components. The isolated NK cells were collected from the interface, washed with PBS, and assessed for purity using the Vi‐Cell counter. The same process was done in replicates of 3 from different blood donors.

### 4.2 NK Cell Isolation from Tonsils

Human tissues were collected and used in accordance with protocols approved by the Ohio State University Institutional Review Board. Donor consent was acquired when deemed appropriate according to the approved Ohio State University Institutional Review Board protocol (IRB #2003H0194). Human pediatric tonsils were obtained fresh following overnight delivery from the Vanderbilt University Division of the Cooperative Human Tissue Network (CHTN) (Nashville, TN). Human innate lymphoid cells (ILCs) were enriched from fresh tonsil tissue. Single‐cell suspensions from fresh human pediatric tonsils were generated by dissociation via a GentleMACS Dissociator (Miltenyi Biotec) according to the manufacturer's instructions. Cells were resuspended in FBS (Sigma‐Aldrich) with leukocyte‐depleted RBCs and a custom human NK cell enrichment RosetteSep reagent containing a mixture of bivalent antibodies against glycophorin A and CD3, CD4, CD19, CD20, CD36, CD66b, and CD123 (STEMCELL Technologies). Cells were mixed with RBCs and RosetteSep reagent, incubated on a nutator for 20 min at room temperature, diluted in PBS (Thermo Fisher Scientific), layered over Ficoll‐Paque PLUS (GE Healthcare), and centrifuged at 2000 rpm for 20 min at room temperature with the break off. The monolayers were harvested, and the residual RBCs were lysed using eBioscience 1 X RBC Lysis Buffer (Thermo Fisher Scientific). From the tonsillar ILC‐enriched cell suspension, ILCs were further enriched using a FACSAria II cell sorter (BD Biosciences). ILCs were sorted as viability dye (Sytox) negative, lineage (CD3, CD4, CD5, CD14, CD19, CD20, CD123, FcER1a, TCRab, TCRgd) negative, and CD45^+^. Cells were sorted into RPMI + 10% FBS and 1% antibiotic‐antimycotic.

### 4.3 Single Cell RNA‐seq/CITE‐seq and Processing

For CITE‐seq, cells from different donors were first labeled with hash tag oligos (HTO) for 20 min on ice, washed once, and then pooled together at equal numbers. Next, TotalSeq A (Biolegend) antibodies containing antibody‐derived tags (ADT) were diluted in PBS‐4% FBS, then used to label cells on ice for 20 min. Cells were then washed twice and processed for single‐cell expression analysis using the 10× Genomics kit. Libraries were amplified for 12 cycles before SPRI bead cleanup and analysis on a tapestation device. Normalized libraries were sequenced on a NovaSeq 6000 S4 flowcell to a depth of 50,000 reads per cell. Downstream analysis was done using Seurat (v. 4.0.3), excluding cells with a high mitochondrial read percentage (>5%) or doublets (>5000 genes per cell).

### 4.4 Single Nuclei ATAC‐seq (snATAC‐seq)

Enriched NK cells from three different donors were processed separately and lysed into single nuclei according to the 10x Genomics snATAC‐seq protocol, targeting 5000–10,000 nuclei for capture. Nuclei were processed following the 10X Genomics snATAC‐seq User Guide, with each sample loaded into a single capture lane. The transposition and nuclei partitioning steps were completed successfully, yielding a uniform emulsion. The GEM incubation PCR was conducted overnight. Library preparation and quality control were subsequently performed according to the 10X Genomics scATAC‐seq protocol.

### 4.5 snATAC‐seq Analysis

Fastq files were demultiplexed and aligned to the GRCh38 human reference genome using the 10x cloud system Ranger, the Cell Ranger Count v7.1.0. The snATAC‐seq data processing pipeline, including quality control, genome alignment, peak calling, normalization, and UMAP clustering, was carried out using the Signac and Seurat tools. Cells were filtered to remove outliers for these metrics: TSS enrichment >2, nucleosome signal <4, and blacklist ratio <0.05. Reads were filtered to remove any contigs or repeats. Peaks under 3 kb were removed, and only cells with more than 200 unique fragments were included. Peak annotation was performed using the reference package from 10x Genomics GRCh38‐2020‐A.

### 4.6 Online Data Acquisition

#### 4.6.1 Bulk RNA‐seq, ATAC‐seq, and ChIP‐seq

Online data mining was current as of 02/2025. Processed ATAC‐ and H3K27ac ChIP‐seq was downloaded from ChIP‐ATLAS using the accession code GSE112813. CTCF ChIP‐seq in NK cells was downloaded from ENCODE using accession number ENCSR856TKC. STAT3 CHIP SEQ. PRDM1 ChIP‐seq in human NK cells was acquired from SRX24830708. Bulk ATAC‐seq of human NK cells was acquired from Holmes et al. [17] through the European Genome‐Phenome Archive (EGA) Dataset ID: EGAD00001008449.

### 4.7 ASAP‐seq

ASAP‐seq of human NK cells was acquired from Rückert et al. [18] through the Gene Expression Omnibus under accession code GSE197037 and analyzed similarly to snATAC‐seq.

### 4.8 Hi‐C Analysis

Hi‐C files were retrieved from the ENCODE project ENCSR538WAP for human neuronal tissue, ENCSR321BHC for human CD8 T cells, and ENCSR971CJS for human NK cells. To visualize the Hi‐C plots and analyze differences between cell types, the 3D genome browser was used [54].

### 4.9 Genome‐Wide Association Studies Analysis

Genome‐wide association studies (GWAS) data for the *NCAM1* locus were downloaded from the UCSC genome browser. Depending on the trait associated with the SNP, SNPs were segregated into immune, psychological, or other and saved as bed files. Bed files were then loaded into IGV to visualize SNP positions in the *NCAM1* locus.

### 4.10 GFP Reporter Assay of NCAM1 Enhancer

One hundred one base pairs (bp) of the NCAM1 DRE (chr11:112,879,032–112,879,132) with and without enhancer were cloned upstream of the CMV promoter next to a GFP reporter gene. Another 101 bp region (chr11:112,942,615–112,942,714) upstream of the NCAM1 that was not accessible in accessible in NK cells was used as a control region and cloned into the same GFP plasmid construct. Plasmids were nucleofected into the YTS cell line. 500,000 cells were nucleofected with 250 ng of plasmid in 3 mL RPMI media. After 45 min, 1.5 mL of cells is taken and stimulated with a combination of 1 unit per mL of IL15, IL18, IL12, and IL21. Twenty hours after nucleofection, cells were stained with viability dye (live/dead BV510). Flow cytometry using Cytek Aurora 5 laser was used to detect GFP expression in viable nucleofected YTS cells. Paired *T*‐test for each replicate was used to measure statistical significance.

## Author Contributions

Mariam A. Salem and Patrick L. Collins wrote the manuscript. Mariam A. Salem, Aditi Varkey, Matthew D. Estrada, Kruthika Sharma, Caprice D. Eisele, and Nitin Chakravarti performed experiments. Mariam A. Salem and Patrick L. Collins analyzed data. Patrick L. Collins and Bethany L. Mundy‐Bosse acquired funding. Patrick L. Collins, Bethany L. Mundy‐Bosse, Aharon G. Freud, and Dean A. Lee supervised. All authors reviewed and edited the manuscript.

## Conflicts of Interest

The authors declare no conflicts of interest.

## Supporting information




**Supporting File**: eji70142‐sup‐0001‐SuppMat.pdf.

## Data Availability

The scATAC‐seq of human peripheral NK cells from three donors are deposited in the Gene Expression Omnibus under accession code GSE297712. CITE‐seq is deposited as well under accession code GSE298188.
