# Supplementary material for: Gene Regulatory Programs of NK Cells Show That NCAM1 (CD56) and KIRs Are Controlled by Genetically Polymorphic Distal Regulatory Elements
Source: Eur J Immunol. 2026 Feb 4;56(2):e70142. doi: 10.1002/eji.70142 (PMC12869470; doi:10.1002/eji.70142)
Supplement: Supplementary file 1 — Supporting File: eji70142‐sup‐0001‐SuppMat.pdf. [file EJI-56-e70142-s001.pdf]

Supplementary Table 1

	Location	*SNP*	*Minor allele frequency (MAF)*	*P-value*	*Heritability explained*	*Effect*
NCAM1	**11:112749800**	**rs77291736**	**0.109**	**6.93E-155**	**0.202**	**-1.076**
NCAM1	**11:112751407**	**rs77738700**	**0.107**	**9.39E-71**	**0.097**	**-0.748**
CCNC	**6:100020651**	**rs181878688**	**0.035**	**7.58E-35**	**0.048**	**0.915**
FCGR3A(CD16)	**1:161508763**	**rs10919544**	**0.453**	**2.40E-20**	**0.027**	**-0.256**
NCAM1	**11:112931625**	**rs1836798**	**0.214**	**9.46E-17**	**0.022**	**0.276**
NCAM1	**11:112751974**	**rs78358948**	**0.110**	**1.17E-15**	**0.021**	**-0.340**
FCGR3A(CD16)	**1:161536758**	**rs147640330**	**0.245**	**1.79E-14**	**0.020**	**0.240**

Figure S1

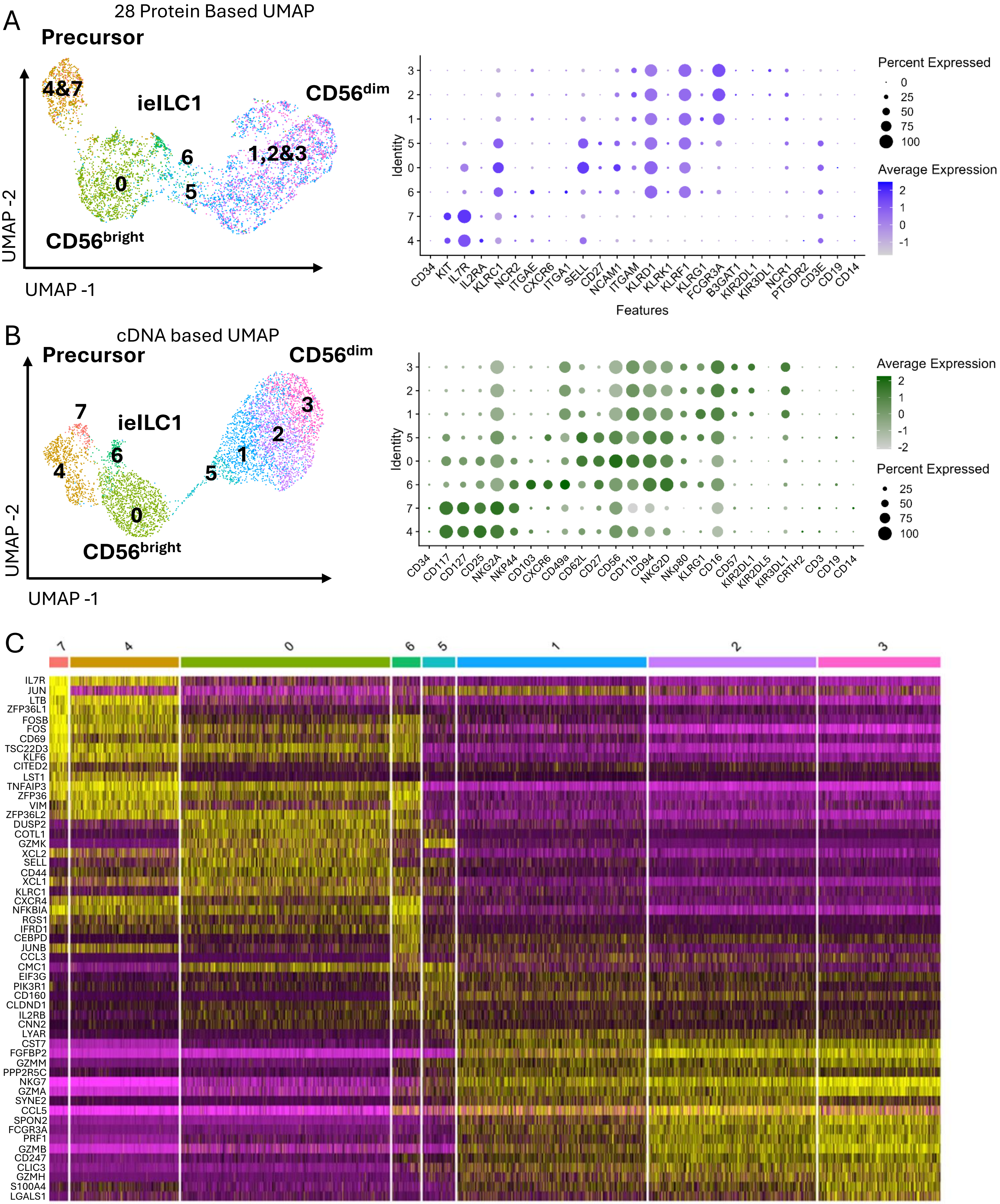
A
28 Protein Based UMAP
Precursor
4&7
ielLC1
CD56dim
6
1,2&3
0
5
CD56bright
UMAP -2
UMAP -1
Percent Expressed
0
25
50
75
100
Average Expression
2
1
0
-1
Identity
3
2
1
5
0
6
7
4
CD34
KIT
IL7R
IL2RA
KLRC1
NCR2
ITGAE
CXCR6
ITGA1
SELL
CD27
NCAM1
ITGAM
KLRD1
KLRK1
KLRF1
KLRG1
FCGR3A
B3GAT1
KIR2DL1
KIR3DL1
NCR1
PTGDR2
CD3E
CD19
CD14
Features
B
cDNA based UMAP
Precursor
CD56dim
7
ielLC1
3
2
6
1
4
5
0
CD56bright
UMAP -2
UMAP -1
Average Expression
2
1
0
-1
-2
Percent Expressed
25
50
75
100
Identity
3
2
1
5
0
6
7
4
CD34
CD117
CD127
CD25
NKG2A
NKP44
CD103
CXCR6
CD49a
CD62L
CD27
CD56
CD11b
CD94
NKG2D
NKp80
KLRG1
CD16
CD57
KIR2DL1
KIR2DL5
KIR3DL1
CRTH2
CD3
CD19
CD14
C
7
4
0
6
5
1
2
3
IL7R
JUN
LTB
ZFP36L1
FOSB
FOS
CD69
TSC22D3
KLF6
CITED2
LST1
TNFAIP3
ZFP36
VIM
ZFP36L2
DUSP2
COTL1
GZMK
XCL2
SELL
CD44
XCL1
KLRC1
CXCR4
NFKBIA
RGS1
IFRD1
CEBPD
JUNB
CCL3
CMC1
EIF3G
PIK3R1
CD160
CLDND1
IL2RB
CNN2
LYAR
CST7
FGFBP2
GZMM
PPP2R5C
NKG7
GZMA
SYNE2
CCL5
SPON2
FCGR3A
PRF1
GZMB
CD247
CLIC3
GZMH
S100A4
LGALS1

Figure S2

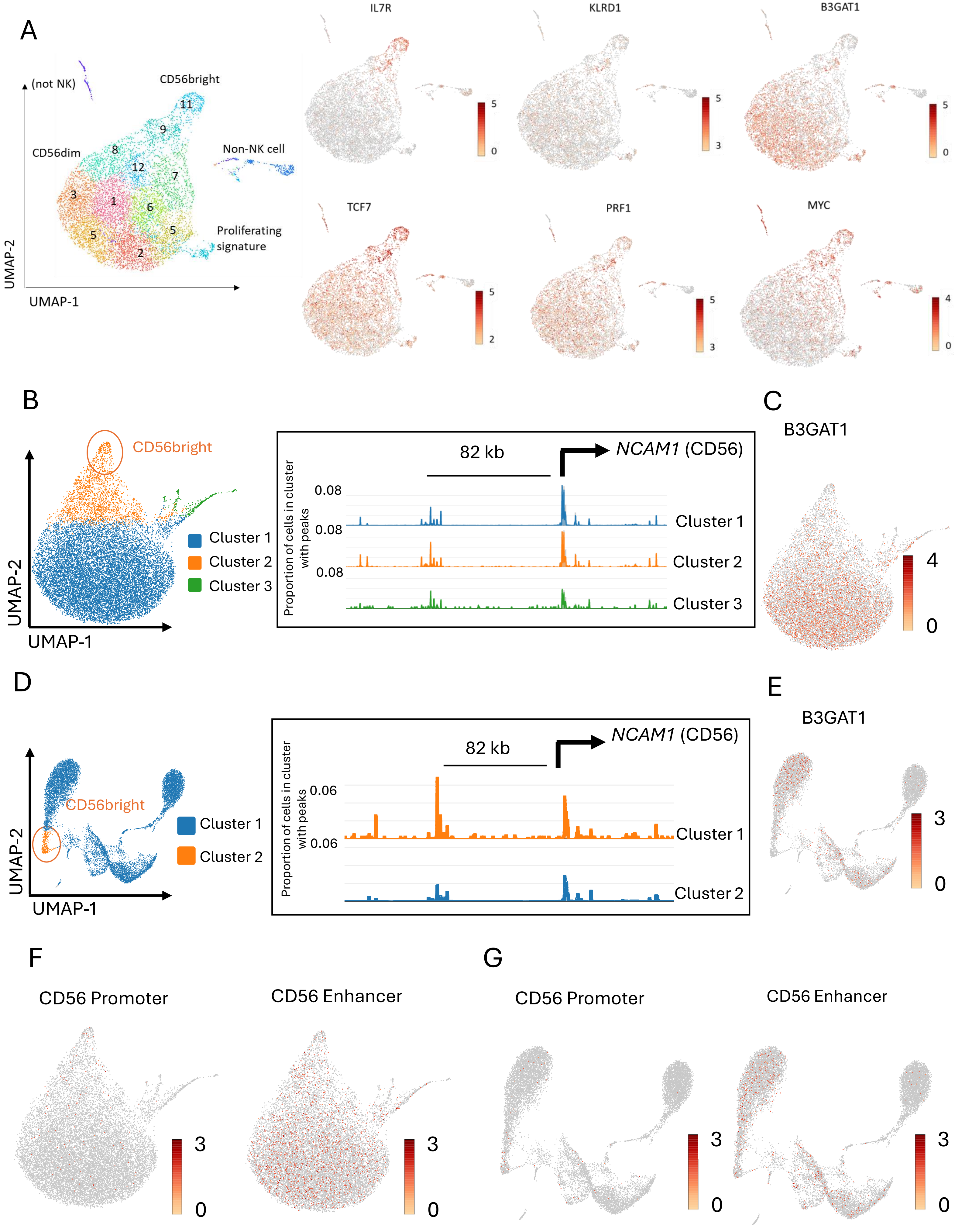

A
IL7R
KLRD1
B3GAT1
(not NK)
CD56bright
CD56dim
Non-NK cell
Proliferating signature
UMAP-2
UMAP-1
TCF7
PRF1
MYC
B
CD56bright
Cluster 1
Cluster 2
Cluster 3
UMAP-2
UMAP-1
82 kb
NCAM1 (CD56)
Proportion of cells in cluster with peaks
0.08
C
B3GAT1
D
CD56bright
Cluster 1
Cluster 2
82 kb
NCAM1 (CD56)
0.06
E
B3GAT1
F
CD56 Promoter
CD56 Enhancer
G
CD56 Promoter
CD56 Enhancer

Figure S3

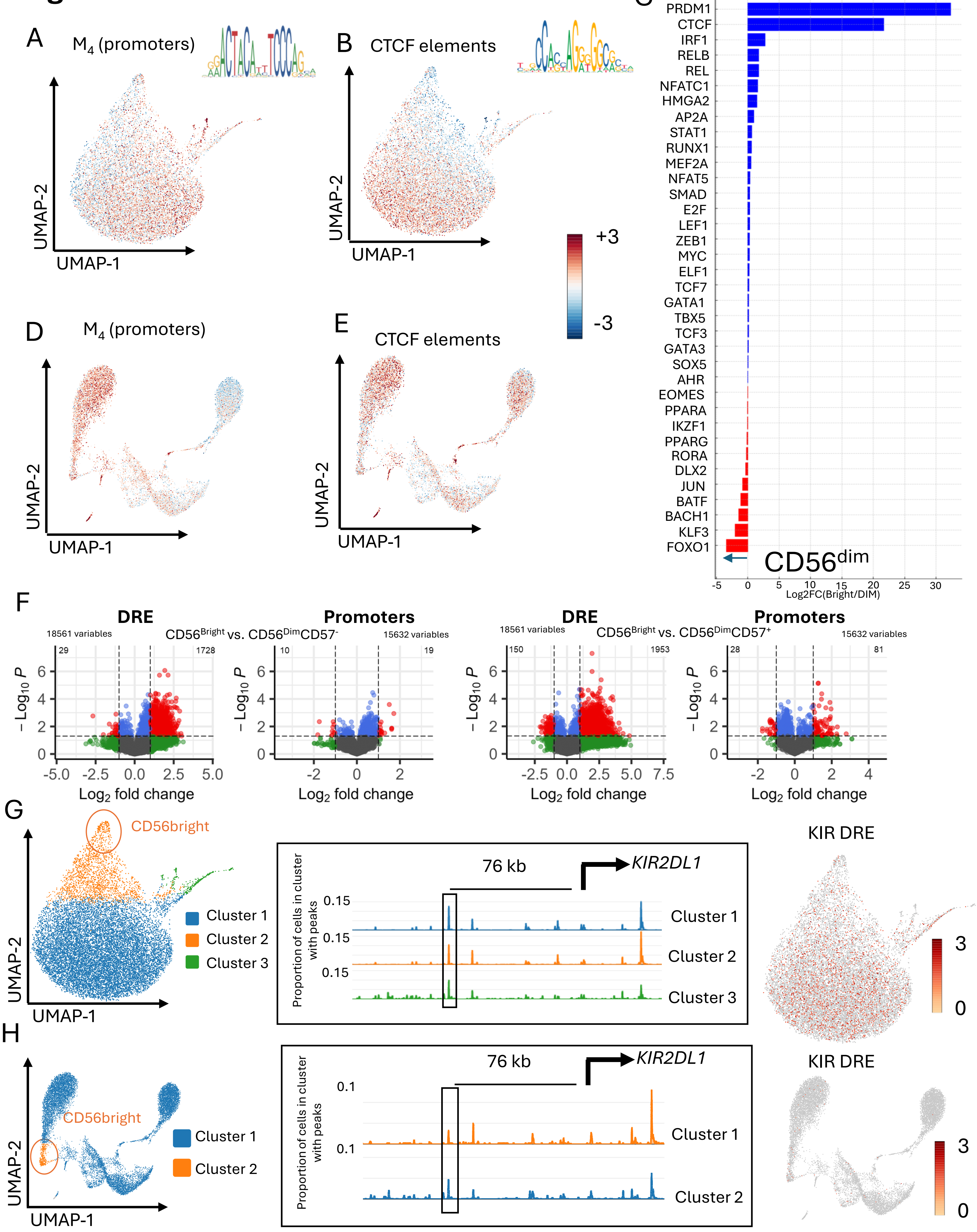

A
M4 (promoters)
UMAP-2
UMAP-1
B
CTCF elements
UMAP-2
UMAP-1
+3
-3
C
CD56bright
PRDM1
CTCF
IRF1
RELB
REL
NFATC1
HMGA2
AP2A
STAT1
RUNX1
MEF2A
NFAT5
SMAD
E2F
LEF1
ZEB1
MYC
ELF1
TCF7
GATA1
TBX5
TCF3
GATA3
SOX5
AHR
EOMES
PPARA
IKZF1
PPARG
RORA
DLX2
JUN
BATF
BACH1
KLF3
FOXO1
CD56dim
-5 0 5 10 15 20 25 30
Log2FC(Bright/DIM)
D
M4 (promoters)
UMAP-2
UMAP-1
E
CTCF elements
UMAP-2
UMAP-1
F
DRE
Promoters
18561 variables
CD56Bright vs. CD56DimCD57-
15632 variables
29
1728
10
19
DRE
Promoters
18561 variables
CD56Bright vs. CD56DimCD57+
15632 variables
150
1953
28
81
−Log10 P
Log2 fold change
G
CD56bright
Cluster 1
Cluster 2
Cluster 3
UMAP-2
UMAP-1
76 kb
KIR2DL1
Proportion of cells in cluster with peaks
0.15
0.15
0.15
Cluster 1
Cluster 2
Cluster 3
KIR DRE
3
0
H
CD56bright
Cluster 1
Cluster 2
UMAP-2
UMAP-1
76 kb
KIR2DL1
Proportion of cells in cluster with peaks
0.1
0.1
Cluster 1
Cluster 2
KIR DRE
3
0

Figure S4

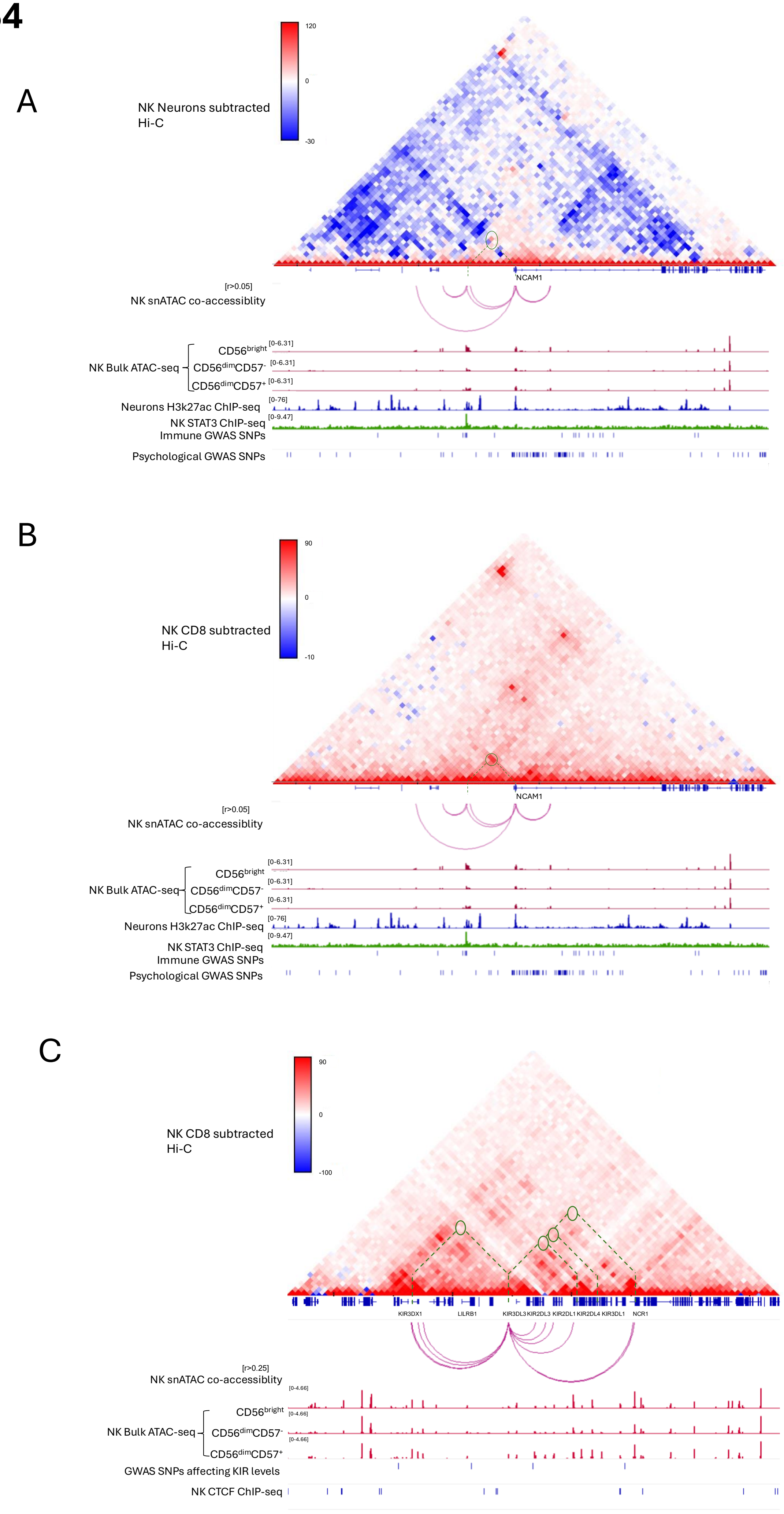
A
NK Neurons subtracted Hi-C
120
0
-30
NCAM1
[r>0.05]
NK snATAC co-accessiblity
NK Bulk ATAC-seq
CD56bright [0-6.31]
CD56dimCD57- [0-6.31]
CD56dimCD57+ [0-6.31]
Neurons H3k27ac ChIP-seq [0-76]
NK STAT3 ChIP-seq [0-9.47]
Immune GWAS SNPs
Psychological GWAS SNPs
B
NK CD8 subtracted Hi-C
90
0
-10
NCAM1
[r>0.05]
NK snATAC co-accessiblity
NK Bulk ATAC-seq
CD56bright [0-6.31]
CD56dimCD57- [0-6.31]
CD56dimCD57+ [0-6.31]
Neurons H3k27ac ChIP-seq [0-76]
NK STAT3 ChIP-seq [0-9.47]
Immune GWAS SNPs
Psychological GWAS SNPs
C
NK CD8 subtracted Hi-C
90
0
-100
KIR3DX1
LILRB1
KIR3DL3
KIR2DL3
KIR2DL1
KIR2DL4
KIR3DL1
NCR1
[r>0.25]
NK snATAC co-accessiblity
NK Bulk ATAC-seq
CD56bright [0-4.66]
CD56dimCD57- [0-4.66]
CD56dimCD57+ [0-4.66]
GWAS SNPs affecting KIR levels
NK CTCF ChIP-seq
